# Quality of life, distress, and posttraumatic growth 5 years after colorectal cancer diagnosis according to history of inpatient rehabilitation

**DOI:** 10.1007/s00432-021-03865-3

**Published:** 2021-12-07

**Authors:** Sophie Scherer-Trame, Lina Jansen, Lena Koch-Gallenkamp, Volker Arndt, Jenny Chang-Claude, Michael Hoffmeister, Hermann Brenner

**Affiliations:** 1 Division of Preventive Oncology, German Cancer Research Center (DKFZ) and National Center for Tumor Diseases (NCT), Im Neuenheimer Feld 581, 69120 Heidelberg, Germany; 2 Medical Faculty Heidelberg, University of Heidelberg, Heidelberg, Germany; 3 Division of Clinical Epidemiology and Aging Research, German Cancer Research Center (DKFZ), Heidelberg, Germany; 4 Epidemiological Cancer Registry Baden-Württemberg, German Cancer Research Center (DKFZ), Heidelberg, Germany; 5 Unit of Cancer Survivorship, Division of Clinical Epidemiology and Aging Research, German Cancer Research Center (DKFZ), Heidelberg, Germany; 6 Division of Cancer Epidemiology, German Cancer Research Center (DKFZ), Heidelberg, Germany; 7 German Cancer Consortium (DKTK), German Cancer Research Center (DKFZ), Heidelberg, Germany

**Keywords:** Inpatient rehabilitation, Colorectal cancer, Cancer survivorship, Health-related quality of life, Distress, Posttraumatic growth

## Abstract

**Purpose:**

In Germany, almost every other colorectal cancer (CRC) patient undergoes inpatient cancer rehabilitation (ICR), but research on long-term outcomes is sparse. We aimed to assess health-related quality of life (HRQOL), distress, and posttraumatic growth among former rehabilitants and non-rehabilitants as well as respective differences and to estimate disease-related quality of life deficits in both groups.

**Methods:**

HRQOL (EORTC-QLQ-C30/CR29), distress (QSC-R10), and posttraumatic growth (PTGI) were assessed according to past ICR in patients 5-year post-CRC-diagnosis in the German DACHS study. Least square mean differences in HRQOL scores and elevated distress levels (QSC-R10 > 14 points) by ICR were estimated by confounder-adjusted linear and logistic regression, respectively. Differences in PTGI scales were tested for statistical significance. EORTC-QLQ-C30 reference scores from population controls were accessed from the LinDE study to estimate disease-related deficits in both treatment groups.

**Results:**

49% of the included 1906 CRC survivors had undergone ICR. Rehabilitants reported lower HRQOL scores than non-rehabilitants in several dimensions of the EORTC-QLQ-C30/CR29. Differences were pronounced among younger survivors (< 70 years). In younger survivors, past ICR also predicted elevated distress. However, rehabilitants showed higher posttraumatic growth. When compared to 934 population controls, non-rehabilitants and older rehabilitants reported HRQOL scores (EORTC-QLQ-C30) similar to controls except higher levels of bowel dysfunctions, whereas younger rehabilitants experienced deficits regarding most scales (13/15).

**Conclusion:**

Our findings suggest a high disease burden 5 years after diagnosis in particular among younger CRC survivors who had undergone ICR. Observed HRQOL deficits are possibly linked to the initial indication for ICR and rehabilitants may benefit from effective follow-up concepts after ICR.

**Supplementary Information:**

The online version contains supplementary material available at 10.1007/s00432-021-03865-3.

## Introduction

Colorectal cancer (CRC) is among the most common cancers globally with approximately 1.9 million new cases in 2020 (Sung et al. 2021). Due to population growth and demographic aging, numbers of new cases are estimated to even increase to more than 2.4 million by 2035 (Douaiher et al. 2017). Prognosis has improved during the last decades in many Western countries (Allemani et al. 2018; Brenner et al. 2012). Consequently, the implementation of cancer rehabilitation and survivorship care has gained importance. CRC survivors have been found to experience poorer health-related quality of life (HRQOL) than the general population, involving bowel disorders, hampered social participation, and financial problems (Arndt et al. 2017; Caravati-Jouvenceaux et al. 2011; Jansen et al. 2011a, b; Thong et al. 2019). Moreover, the disease and its consequences can affect CRC survivors’ mental wellbeing profoundly (Custers et al. 2016; Jansen et al. 2011a). About every third cancer survivor seems to experience cancer-related psychosocial distress in daily life after treatment period (Herschbach et al. 2020). Nevertheless, coping with the potential life-threatening cancer disease and its consequences may also lead to positive psychological changes regarding interpersonal orientation, life perspective, and self-perception known as posttraumatic growth (Ochoa Arnedo et al. 2019; Salsman et al. 2009; Tedeschi and Calhoun 1996).

In Germany, cancer rehabilitation is predominately offered as multi-professional inpatient programs in specialized rehabilitation clinics, which provide post-acute care directly after primary cancer treatment as well as follow-up care mainly within the first year after completion of treatment. Rehabilitation programs usually last 3 weeks, and consist of closely coordinated interventions such as nutrition counseling, physical and exercise therapy and stoma care, patient education, and psychological support. In addition to the reduction of physical disabilities, rehabilitation programs aim to impart knowledge about the disease and sociomedical aspects and to teach self-management strategies to cope with the disease and disabilities in daily life (Bilsing et al. 2015).

According to the German CRC S3-Guideline, all CRC patients with sufficient capacity to tolerate rehabilitation treatment should receive an offer for cancer rehabilitation (German Guideline Program in Oncology 2019). In addition to capacity, preconditions set by healthcare payers include patients’ need for rehabilitation, e.g., cancer or therapy-related physical or mental impairments interfering with daily life, professional or social life, and a positive rehabilitation prognosis. Results from a patient survey indicate that applications for cancer rehabilitation at the respective payers are usually successful (Deck et al 2019). The German Pension Insurance acts as a mayor healthcare payer for cancer rehabilitation. In contrast to non-cancer-specific indications, it funds rehabilitation measures not only for working but also for a large proportion of retired patients next to statutory health insurances. About 44–50% of the CRC patients undergo inpatient cancer rehabilitation (ICR) (Deck et al. 2019; Nowossadeck and Barnes 2016; Scherer-Trame et al. 2021; Waldmann et al. 2007). Reasons for non-utilization of ICR include no perceived disability, family ties, job commitments, and the wish to return to daily life (Deck et al. 2019).

Despite the high utilization of ICR among CRC patients, evidence of effectiveness of such treatment is limited (Scherer et al. 2021). Previous studies have observed improvements in several dimensions of HRQOL suggesting largest benefits with respect to global quality of life, physical, emotional, social functioning, and fatigue (Lamprecht et al. 2017; Riedl et al. 2017; Singer and Schulte 2009). However, these studies were of uncontrolled design (i.e., no inclusion of non-rehabilitants), and it is unknown if the improvements persist over time or wane after ICR as seen for other health-related outcomes (Allgayer et al. 2005, 2012; Biskup et al. 1994; Mehnert et al. 2013). When compared to reference values from the general population, CRC rehabilitants showed deficits in most dimensions of HRQOL before ICR, which may reflect the indication for treatment (Riedl et al. 2017; Singer and Schulte 2009). Due to the short observation periods of the respective studies, little is known about long-term HRQOL deficits in CRC survivors, who had undergone ICR. Moreover, study findings regarding the reduction of psychological distress during or after ICR are inconsistent (Klocker et al. 2018; Lamprecht et al. 2017; Riedl et al. 2017; Ross et al. 2015). Since rehabilitation research mainly focuses on health outcomes among rehabilitants, potential long-term HRQOL deficits in cancer survivors, who have not undergone ICR, are largely unknown.

Thus, we aimed to assess HRQOL, cancer-related distress, and posttraumatic growth among former rehabilitants and non-rehabilitants 5 years after CRC diagnosis, and to estimate respective differences between both treatment groups. We furthermore aimed to assess HRQOL deficits among former rehabilitants and non-rehabilitants in comparison with population controls.

## Materials and methods

### Study design and population

Our analysis is based on CRC patients recruited in the DACHS *(Darmkrebs: Chancen der Verhütung durch Screening*) study, an ongoing population-based case–control study with long-term follow-up of CRC cases conducted in the Rhine-Neckar region of Germany. Details on the study design have been reported elsewhere (Brenner et al. 2011; Hoffmeister et al. 2015). German-speaking patients, at least 30 years of age, from the study region with first diagnosis of CRC (C18-C20, International Classification of Disease, revision 10), who are able to participate in a one-hour interview are eligible for the study. Patients are recruited in 22 cooperating hospitals by treating physicians, typically during the first hospitalization due to CRC. The study was approved by the ethic committees of the state medical board of Baden-Wuerttemberg and Rhineland-Palatinate as well as the Medical Faculty of Heidelberg.

### Data collection and follow-up

Trained interviewers obtain sociodemographic data, lifestyle-related information, and the medical history of the participants during a standardized face-to-face interview at baseline close to initial diagnosis. For CRC patients, collected discharge letters and pathology reports provide tumor- and surgery-related information. Three years after diagnosis, therapy-related information including the utilization of ICR (yes/no) and name of rehabilitation clinic is given retrospectively by the attending general practitioners and/or oncologist. The physicians fill out questionnaires based on patient records and provide medical reports. Five years after diagnosis, participants receive a follow-up questionnaire by mail to assess HRQOL and further self-reported outcomes. If participants refuse to fill out the questionnaire but are willing to answer a short version of the questionnaire including only personal and medical information, the short version of the questionnaire is sent out. Throughout the study, patient’s vital status registered by the population registries is checked regularly. For this analysis, an additional retrospective collection of rehabilitation discharge letters via rehabilitation clinics and treating physicians was conducted for a subset of rehabilitants to approximate timing of ICR after CRC diagnosis.

### Inclusion and exclusion criteria

For the current analysis, we included CRC patients diagnosed in 2005–2013 who were still alive 5 years after diagnosis and who participated in the 5-year follow-up (5YFU) in 2010–2018. We excluded CRC survivors with unavailable information on utilization of ICR or who underwent outpatient rehabilitation care only from the analysis. We furthermore excluded participants who provided a short version of the follow-up questionnaire only.

### Assessment of HRQOL, distress, and posttraumatic growth

HRQOL was measured with the EORTC-QLQ-C30 questionnaire Version 3.0 (Aaronson et al. 1993) at 5YFU. The instrument was developed by the European Organization for Research and Treatment of Cancer and includes one global quality-of-life scale, five functioning scales (physical, cognitive, role, emotional, and social), and nine symptom scales (insomnia, fatigue, pain, dyspnea, constipation, diarrhea, appetite loss, nausea and vomiting, and financial impact). Participants were asked to rate each item on a Likert scale from 1 (“not at all”) to 4 (“very high”). Scores for every scale were calculated and linearly transformed to a 0–100 scale according to the scoring manual (Fayers et al. 2001). Higher scores on the global and functioning scales reflect better HRQOL, whereas higher scores on the symptom scales indicate higher symptom burden.

In addition to the core questionnaire, the CRC-specific submodule (EORTC-QLQ-CR29) was administered to assess 4 CRC-related functioning (future perspective, body image, weight, and sexual interest) and 18 symptom scales (Gujral et al. 2007; Whistance et al. 2009). Scoring and interpretation of scores were analogues to the core questionnaire (Fayers et al. 2001).

Cancer-related distress was measured with the Questionnaire on Stress in Cancer Patients (QSC-R10) (Book et al. 2011). Its ten items address the impact of cancer-related stressors including psychosomatic complaints, fears, information deficits, everyday life restrictions, and social strains. The instrument is regularly used as a screening tool in clinical practice to identify psychosocial supportive needs in cancer patients. Based on the total score, we classified a value above 14 points as a positive screening result (Book et al. 2011) reflecting an elevated level of distress.

Posttraumatic growth was measured with selected scales of the Posttraumatic Growth Inventory (Tedeschi and Calhoun 1996). The instrument assesses positive outcomes as consequences of a traumatic event. The 5YFU included all scale-specific items for the factors: new possibilities, spiritual change, and appreciation of life. Each factor scale ranges from 0 to 5. Higher scores imply stronger posttraumatic growth in the respective area (Tedeschi and Calhoun 1996).

### Reference HRQOL scores from the general population

To estimate HRQOL deficits among rehabilitants and non-rehabilitants in comparison to population controls, we included data from the LinDE study. The LinDE study was a cross-sectional survey conducted in 2013/2014 to obtain normative HRQOL data, among other self-reported health outcomes, in a randomly selected sample of the German population for comparisons with cancer patients and survivors. Age- and sex-based sampling was performed by the regional municipal offices. Potential participants, aged 18 and above, received study information and the questionnaire including the EORTC-QLQ-C30 instrument by mail. In contrast to most published aggregated normative HRQOL data (Nolte et al. 2019; Waldmann et al. 2013), the LinDE study was a nationwide study, oversampling the older age groups to guarantee a sufficient sample size for comparisons with cancer survivors in those age groups most affected by cancer. Moreover, data are available on an individual level. The response rate was 29%. Further study details are published elsewhere (Arndt et al. 2017). Based on the age- and sex-specific distribution of the CRC rehabilitants in the DACHS study, we performed a frequency distribution matching and drew a sample of comparable population controls after excluding controls outside of rehabilitants’ age range or with a prior history of CRC.

### Statistical analysis

#### Patient-reported outcomes according to utilization of ICR among CRC survivors

All patient-reported outcomes were analyzed for younger and older survivors (i.e., age at follow-up < / ≥ 70 years) separately. Adjusted means for all HRQOL scales (EORTC-QLQ-C30 /CR29) were computed using multivariable linear regression models to describe and compare HRQOL among survivors who had undergone ICR (rehabilitants) and those who had not undergone ICR (non-rehabilitants). Models were stratified for age at follow-up, and adjusted for baseline variables including sex, education, partnership, employment status, health insurance type, UICC stage, tumor location, laparoscopic surgery, radiotherapy, chemotherapy, ostomy, comorbidities (Charlson comorbidity index (Charlson et al. 1987)), and prediagnosis physical activity level (categorization of covariates see Table 1). We selected the potential confounders based on a previous analysis of determinants of receiving ICR (Scherer-Trame et al. 2021) and on predictors of HRQOL (Bours et al. 2016). Differences between rehabilitants and non-rehabilitants were quantified by adjusted least square mean differences (LSMD).

**Table 1:** Characteristics of colorectal cancer survivors according to rehabilitation treatment

Sociodemographic, clinical, and lifestyle characteristics*	Total		Rehabilitants		Non-rehabilitants		*p *value**
	*N* = 1906 (100%)		*n* = 934 (49.0%)		*n* = 972 (51.0%)		
	*N*	Col %	*n*	Col %	*n*	Col %	
Age at diagnosis (in years)							
< 50	123	6.5	73	7.8	50	5.1	< .0001^a^
50–64	630	33.1	342	36.6	288	29.6	
65–79	981	51.5	432	46.3	549	56.5	
≥ 80	172	9.0	87	9.3	85	8.7	
Sex							
Women	750	39.4	396	42.4	354	36.4	0.0076^b^
Men	1156	60.7	538	57.6	618	63.6	
Years of school education^1^							
≤ 9	1186	62.4	583	62.4	603	62.0	0.1106^b^
10	352	18.5	185	19.8	167	17.2	
12/13	364	19.1	163	17.5	201	20.7	
Employment status^1^							
Employed	493	26.0	296	31.7	197	20.3	< .0001^a^
Self-employed	56	3.0	17	1.8	39	4.0	
Unemployed	28	1.5	19	2.0	9	0.9	
Retired	1153	60.8	520	55.7	633	65.1	
Housewife/-man	168	8.9	79	8.5	89	9.2	
Living in a partnership^1^							
Yes	1523	80.1	725	77.6	798	82.1	0.0187^b^
No	379	19.9	206	22.1	173	17.8	
Health insurance							
Statutory	1356	71.1	691	74.0	665	68.4	< .0001^a^
Private	188	9.9	62	6.6	126	13.0	
Unknown	362	19.0	181	19.4	181	18.6	
Cancer site							
Colon	1127	59.1	557	59.6	570	58.6	0.7097^b^
Rectum	779	40.9	377	40.4	402	41.4	
Tumor stage, UICC^2^							
I	534	28.4	229	24.5	305	31.4	0.0076^a^
II	639	34.0	324	34.7	315	32.4	
III	641	34.0	336	36.0	305	31.4	
IV	68	3.6	36	3.9	32	3.3	
Tumor resection^1^							
Non	3	0.2	2	0.2	1	0.1	0.2433^b^
Open	1582	83.1	783	83.8	799	82.2	
Laparoscopic	318	16.7	146	15.6	172	17.7	
Ostomy^1^							
Yes	613	32.2	320	34.3	295	30.3	0.0699^b^
No	1289	67.8	613	65.6	676	69.5	
Chemotherapy^1^							
Yes	790	41.5	408	43.7	382	39.3	0.0567^b^
No	1115	58.5	525	56.2	590	60.7	
Radiation							
Yes	356	18.7	183	19.6	173	17.8	0.3471^b^
No	1550	81.3	751	80.4	799	82.2	
CCI scores^3^							
0	1185	62.2	597	63.9	588	60.5	0.1543^a^
1	397	20.8	196	21.0	201	20.7	
2	188	9.9	78	8.4	110	11.3	
3 +	136	7.1	63	6.7	73	7.5	
Prediagnosis physical activity level^1,4^							
Low	627	33.1	273	29.2	354	36.4	0.0044^a^
Medium	633	33.4	328	35.1	305	31.4	
High	636	33.5	327	35.0	309	31.8	
Recurrence until 5YFU^1^							
Yes	205	10.8	89	9.5	116	11.9	0.1036^b^
No	1699	89.2	844	90.4	855	88.0	

*CCI* Charlson comorbidity index score; *UICC* International Union against Cancer; *5YFU* 5-year follow-up

*Patient characteristics refer to the time around CRC diagnosis, unless otherwise stated

**P values of the comparison between rehabilitants and non-rehabilitants from Chi-square test of independence (a) or Fisher’s exact test (b)

^1^1–10 missings

^2^24 missings

^3^The Charlson comorbidity index (CCI) (Charlson et al. 1987) was used to score reported comorbidities and group participants into four groups from no (0) to severe comorbidity (3 +)

^4^Prediagnosis physical activity level was assessed as metabolic equivalents hours per week and categorized by tertiles according to patient-reported type of activity and averages of minutes spent: Low: Q1 < 88.2; medium: Q2 = 88.2–153.5; high: Q3 ≥ 153.5 MET hours/week

We assessed positivity with respect to cancer-related distress among rehabilitants and non-rehabilitants, and compared it by Chi-square test of independence and multivariable logistic regression models, adjusting for the same baseline variables outlined above for the linear regression models.

To assess potential differences between rehabilitants and non-rehabilitants on single posttraumatic growth scales, means and medians by treatment groups were calculated, and Mann–Whitney *U* test was applied.

#### *Deficits in *HRQOL o*f CRC survivors in comparison to population controls*

We estimated HRQOL deficits assessed with the core questionnaire (EORTC-QLQ-C30) among rehabilitants and non-rehabilitants separately in comparison to population controls by linear regression models. Models were stratified by age at follow-up/survey (< / ≥ 70 years) and adjusted for sex and education. Adjusted means for each group (rehabilitants/non-rehabilitants/controls) were computed and deficits were quantified by adjusted LSMD.

All analyses were performed on SAS version 9.4 (SAS Institute Inc., Cary, NC, USA). Statistical significance was defined by a two-sided *P* < 0.05.

## Results

### DACHS study participants

Among the 2125 CRC survivors who participated in the 5YFU (response rate 86.5%), 154 (7.2%) sent back a short form of the questionnaire only, for 63 participants (3.0%) prior ICR was unknown and two participants (< 0.1%) underwent outpatient rehabilitation only. In the final analyses, we included 1906 survivors (see Supplementary Fig. S1). Sociodemographic and clinical characteristics of survivors according to past utilization of ICR are presented in Table 1. About 11% of the survivors had experienced CRC recurrence until the 5YFU. The mean age of the participants was 66.3 at diagnosis and 71.3 at follow-up (± 10.4). Approximately half (49%) of the participants had undergone ICR. Rehabilitants were slightly younger and more likely to be employed at the time of diagnosis, to have no partner, to have statutory health insurance, and to have higher prediagnosis physical activity level, and were less likely to be diagnosed with cancer stage I compared to non-rehabilitants. Based on available rehabilitation discharge letters for a subset of rehabilitants (53%), time from diagnosis to start of ICR was calculated. The majority of rehabilitants underwent ICR within the first 2 months after CRC diagnosis (median = 1.7 months) and the mean time from diagnosis to start of ICR was 5.2 months (standard deviation = 7.4 months).

### Patient-reported outcomes among rehabilitants and non-rehabilitants

#### HRQOL

Table 2 presents adjusted HRQOL mean scores (EORTC-QLQ-C30) for participants below and above 70 years of age by prior utilization of ICR, and shows LSMD between treatment groups. Among younger CRC survivors, we observed poorer functioning of former rehabilitants in 4/5 scales (cognitive, role, emotional, and social) and higher symptom burden in 4/9 scales (insomnia, fatigue, pain, and dyspnea), in addition to lower global quality of life 5 years after diagnosis. LSMD between younger rehabilitants and younger non-rehabilitants were mostly of moderate size (< 8 points), but always indicating poorer functioning and higher symptom in rehabilitants. Differences in HRQOL scores according to prior utilization of ICR were less pronounced (< 5 points) in older participants and statistically significant only for the following scales: emotional functioning, cognitive functioning, insomnia, fatigue, nausea and vomiting.

**Table 2:** HRQOL (EORTC-QLQ-C30 scores) among rehabilitants and non-rehabilitants stratified by age

	Age at follow-up < 70 years (*n* = 752)			Age at follow-up ≥ 70 years (*n* = 1154)		
	Rehabilitants	Non-rehabilitants	Rehabilitants vs non-rehabilitants	Rehabilitants	Non-rehabilitants	Rehabilitants vs non-rehabilitants
	Mean (SE)^1^	Mean (SE)^1^	Mean Diff. (95% CI)^1^	Mean (SE)^1^	Mean (SE)^1^	Mean Diff. (95% CI)^1^
Functioning scales^2^						
Physical	79.0 (2.5)	81.1 (2.4)	− 2.0 (− 4.9, 0.9)	75.6 (2.8)	76.9 (2.8)	− 1.3 (− 4.2, 1.6)
Role	68.1 (3.9)	74.6 (3.6)	**− 6.5 (− 10.9, − 2.0)**	71.1 (3.7)	74.8 (3.6)	− 3.7 (− 7.5, 0.1)
Emotional	67.7 (3.5)	72.0 (3.3)	**− 4.4 (− 8.4, − 0.4)**	71.9 (2.9)	75.2 (2.9)	**− 3.3 (− 6.3, − 0.3)**
Cognitive	74.5 (3.0)	81.8 (2.8)	**− 7.3 (− 10.8, − 3.8)**	78.1 (2.8)	81.4 (2.8)	**− 3.3 (− 6.2, − 0.4)**
Social	68.6 (4.0)	76.4 (3.7)	**− 7.9 (− 12.4, − 3.3)**	77.3 (3.5)	79.2 (3.4)	− 1.9 (− 5.5, 1.7)
Global QOL	62.4 (3.0)	68.8 (2.8)	**− 6.4 (− 9.9, − 2.9)**	60.5 (2.6)	61.8 (2.5)	− 1.3 (− 3.9, 1.4)
Symptom scales^3^						
Insomnia	38.1 (4.6)	31.6 (4.3)	**6.5 (1.2, 11.7)**	40.3 (3.8)	35.4 (3.7)	**4.9 (0.9, 8.8)**
Fatigue	37.4 (3.6)	32.4 (3.4)	**5.1 (0.9, 9.2)**	41.6 (3.1)	36.8 (3.1)	**4.8 (1.6, 8.0)**
Pain	25.8 (3.9)	19.5 (3.6)	**6.2 (1.8, 10.7)**	28.5 (3.6)	24.9 (3.5)	3.6 (− 0.1, 7.3)
Dyspnea	27.4 (3.7)	25.6 (3.5)	1.8 (− 2.4, 6.1)	27.3 (3.8)	23.9 (3.7)	3.4 (− 0.4, 7.2)
Constipation	13.0 (3.6)	7.7 (3.3)	**5.3 (1.2, 9.4)**	12.5 (3.3)	11.6 (3.2)	0.8 (− 2.5, 4.2)
Diarrhea	28.3 (4.3)	25.0 (4.0)	3.3 (− 1.7, 8.3)	27.6 (3.4)	24.8 (3.3)	2.9 (− 0.6, 6.4)
Appetite loss	12.1 (2.6)	11.2 (2.4)	0.9 (− 2.1, 3.9)	9.6 (2.7)	6.9 (2.6)	2.7 (0.0, 5.4)
Nausea and vomiting	8.9 (1.9)	8.5 (1.8)	0.4 (− 1.8, 2.6)	4.9 (1.5)	3.3 (1.5)	**1.6 (0.0, 3.1)**
Financial impact	28.9 (4.2)	24.5 (3.9)	4.4 (− 0.4, 9.2)	13.3 (2.8)	10.6 (2.8)	2.7 (− 0.2, 5.6)

Significant mean differences are printed in bold

*CI* confidence interval; *Diff.* differences; *SE* standard error

^1^Adjusted for sex, education, partnership, employment status, type of health insurance, UICC stage, tumor location, laparoscopic surgery, radiotherapy, chemotherapy, ostomy, comorbidities, and pre-diagnosis physical activity. Covariates include baseline information

^2^Higher scores indicate better functioning/negative differences indicate poorer functioning compared to respective reference group. Missing values for every scale < 1%

^3^Higher scores indicate higher symptom burden/positive differences indicate higher symptom burden compared to respective reference groups. Missing values for every scale < 1%

We observed similar patterns regarding the CRC-specific HRQOL scores (CR29) (see Table 3). Linear regression models based on younger survivors yielded significant LSMD in almost half of all scales. Results indicated poorer functioning (body image and weight) and higher symptom burden (up to nine points) in particular due to defecation-related problems (stool frequency, fecal incontinence, sore skin, bloated feeling, flatulence, embarrassed by bowel movements, abdominal pain, and buttock pain) in rehabilitants when compared to non-rehabilitants. Among older survivors, results implied higher symptom burden (up to five points) in rehabilitants regarding the following scales: dry mouth, urinary frequency, urinary incontinence, stool frequency, flatulence, embarrassed by bowel movements, buttock pain, and dyspareunia. LSMD were generally less pronounced in older survivors than in younger survivors.

**Table 3:** HRQOL (EORTC-QLQ-CR29 scores) among rehabilitants and non-rehabilitants stratified by age

	Age at follow-up < 70 years (*n* = 752)			Age at follow-up ≥ 70 years (*n* = 1154)		
	Rehabilitants	Non-rehabilitants	Rehabilitants vs non-rehabilitants	Rehabilitants	Non-rehabilitants	Rehabilitants vs non-rehabilitants
	Mean (SE)^1^	Mean (SE)^1^	Mean Diff. (95% CI)^1^	Mean (SE)^1^	Mean (SE)^1^	Mean Diff. (95% CI)^1^
Functioning scales^2^						
Body image^a^	66.3 (3.6)	74.7 (3.4)	**− 8.4 (− 12.6, − 4.2)**	78.3 (2.8)	80.1 (2.7)	− 1.7 (− 4.5, 1.1)
Future perspective^a^	61.4 (4.4)	64.1 (4.1)	− 2.7 (− 7.7, 2.3)	60.7 (3.9)	62.8 (3.8)	− 2.0 (− 6.0, 1.9)
Weight^a^	71.7 (4.4)	77.4 (4.1)	**− 5.7 (− 10.8, − 0.6)**	78.8 (3.6)	81.0 (3.5)	− 2.2 (− 5.9, 1.4)
Sexual interest women^b^	26.1 (7.9)	27.9 (7.9)	− 1.8 (− 9.8, 6.1)	19.1 (5.7)	21.2 (5.7)	− 2.1 (− 7.6, 3.3)
Sexual interest men^b^	54.3 (8.5)	56.9 (8.0)	− 2.6 (− 9.3, 4.0)	47.3 (5.5)	46.9 (5.4)	0.4 (− 4.7, 5.5)
Symptom scales^3^						
Dry Mouth^a^	25.1 (3.8)	22.2 (3.5)	2.9 (− 1.5, 7.3)	29.4 (3.6)	25.3 (3.5)	**4.2 (0.5, 7.8)**
Trouble with taste^a^	14.4 (3.3)	12.3 (3.9)	2.1 (− 1.7, 5.9)	12.1 (2.7)	10.9 (2.6)	1.1 (− 1.6, 3.9)
Hair loss^a^	16.8 (3.1)	16.7 (2.9)	0.1 (− 3.5, 3.7)	14.3 (2.8)	11.9 (2.7)	2.3 (− 0.5, 5.2)
Urinary frequency^a^	31.9 (3.6)	32.4 (3.3)	− 0.6 (− 4.7, 3.6)	47.8 (3.2)	43.5 (3.1)	**4.4 (1.1, 7.6)**
Urinary incontinence^c^	10.4 (3.2)	10.2 (3.0)	0.2 (− 3.5, 3.9)	21.6 (3.3)	15.7 (3.27)	**5.9 (2.5, 9.3)**
Dysuria^a^	1.7 (1.7)	1.0 (1.6)	0.6 (− 1.3, 2.6)	4.0 (1.8)	2.9 (1.7)	1.1 (− 0.7, 2.9)
Stool frequency^c^	37.6 (3.6)	32.6 (3.4)	**4.9 (0.8, 9.1)**	34.6 (2.9)	31.2 (2.8)	**3.3 (0.4, 6.2)**
Fecal incontinence^c^	27.3 (3.6)	22.9 (3.3)	**4.4 (0.3, 8.5)**	26.00 (3.0)	23.4 (3.0)	2.6 (− 0.5, 5.6)
Blood/mucus in stool^a^	5.9 (1.6)	4.5 (1.5)	1.4 (− 0.5, 3.2)	4.0 (1.2)	3.5 (1.2)	0.4 (− 0.8, 1.6)
Sore skin^c^	22.6 (3.8)	17.3 (3.6)	**5.3 (0.9, 9.7)**	25.0 (3.0)	22.4 (3.0)	2.6 (− 0.5, 5.7)
Stoma care problems^a^	26.5 (11.4)	23.9 (11.4)	2.6 (− 9.5, 14.7)	13.8 (16.5)	1.5 (16.0)	12.4 (− 1.9, 26.6)
Bloated feeling^a^	35.5 (4.3)	25.7 (4.1)	**9.8 (4.8, 14.8)**	26.1 (3.5)	22.9 (3.4)	3.2 (− 0.3, 6.8)
Flatulence^c^	41.0 (4.3)	31.2 (4.0)	**9.8 (4.8, 14.8)**	40.8 (3.7)	35.1 (3.6)	**5.8 (2.1, 9.5)**
Embarrassment^c^	29.2 (4.2)	21.1 (3.9)	**8.2 (3.4, 13.0)**	23.5 (3.4)	19.7 (3.3)	**3.7 (0.3, 7.2)**
Abdominal pain^a^	17.9 (3.4)	10.9 (3.2)	**7.1 (3.2, 10.9)**	13.8 (2.6)	11.7 (2.6)	2.2 (− 0.5, 4.9)
Buttock pain^a^	16.6 (3.5)	12.2 (3.3)	**4.4 (0.4, 8.4)**	22.1 (2.7)	19.0 (2.7)	**3.1 (0.3, 5.9)**
Impotence^d^	50.1 (9.9)	47.3 (9.3)	2.8 (− 5.0, 10.5)	61.1 (6.7)	64.8 (6.6)	− 3.7 (− 10.0, 2.6)
Dyspareunia^d^	28.9 (9.4)	21.7 (9.5)	7.3 (− 2.7, 17.2)	2.9 (5.2)	− 2.5 (5.3)	**5.4 (0.3, 10.5)**

Significant mean differences are printed in bold

*CI* confidence interval; *Diff.* differences; *SE* standard error

^1^Adjusted for sex (excluding sex-specific scales), education, partnership, employment status, type of health insurance, UICC stage, tumor location, laparoscopic surgery, radiotherapy, chemotherapy, ostomy (excluding stoma care scale), comorbidities, and pre-diagnosis physical activity. Covariates include baseline information

^2^Higher scores indicate better functioning/negative differences indicate poorer functioning compared to respective reference group

^3^Higher scores indicate higher symptom burden/positive differences indicate higher symptom burden compared to respective reference groups

^a^ < 1.2% missing values within the study cohort

^b^unknown/not applicable 3.0% in men, 18.8% in women

^c^ < 2.5% missing values within the study cohort

^d^unknown/not applicable 6.3% in men, 36.7% in women

#### Distress

Among the younger rehabilitants, 42.1% were screened positive for elevated distress levels, whereas 23.4% were screened positive among the non-rehabilitants 5 years after diagnosis (*p* < 0.001, adjusted odds ratio in the logistic regression: aOR 2.46, 95% CI 1.71–3.54). Among older CRC survivors, proportions of positive screened were similar among rehabilitants (30.2%) and non-rehabilitants (26.6%) (*p* = 0.187, aOR 1.19, CI 0.90–1.58).

#### Posttraumatic growth

Rehabilitants of both age groups reported higher posttraumatic growth in several dimensions compared to non-rehabilitants. Table 4 presents results in detail. For younger rehabilitants, significantly higher growth was observed for the dimensions new possibilities and appreciation of life, and for older rehabilitants, significantly higher growth was observed related to new possibilities and spiritual enhancement.

**Table 4:** Posttraumatic Growth Inventory scores among rehabilitants and non-rehabilitants stratified by age

	Age at follow-up < 70 years (*n* = 752)						Age at follow-up ≥ 70 years (*n* = 1154)					
	Rehabilitants		Non-rehabilitants		Rehabilitants vs non-rehabilitants		Rehabilitants		Non-rehabilitants		Rehabilitants vs non-rehabilitants	
	Mean (SD)	Median	Mean (SD)	Median	*p* *value*^***1***^	Effect size^2^ r	Mean (SD)	Median	Mean (SD)	Median	*p* value^1^	Effect size^2^ r
Posttraumatic Growth Inventory^3^												
New possibilities (0–5)	2.0 (1.1)	1.8	1.6 (1.1)	1.6	< .0001	− 0.16	1.6 (1.1)	1.4	1.4 (1.1)	1.2	0.0011	0.10
Spiritual enhancement (0–5)	1.4 (1.4)	1.0	1.3 (1.4)	1.0	0.0877	− 0.06	1.6 (1.4)	1.5	1.4 (1.4)	1.0	0.0073	0.08
Appreciation (0–5)	3.2 (1.2)	3.3	2.8 (1.3)	3.0	0.0001	− 0.14	2.8 (1.2)	3.0	2.6 (1.4)	2.7	0.0829	0.05

*SD* standard deviation;

^1^Two sided Mann–Whitney *U* test

^2^Effect size estimated by Pearson correlation coefficient *r*

^3^Full questionnaire was not administered. Presented single scales (missing values < 2.7% within the study cohort) included all scale-specific items

### ***Deficits i***n HRQOL ***of CRC survivors in comparison to population controls***

#### LinDE study: population controls

From 2849 eligible LinDE participants, we excluded 406 whose age was outside the rehabilitants’ age range at follow-up (39–96 years) and 32 patients with a history of CRC. After the sex- and age-based frequency distribution matching, HRQOL data from 934 population controls remained for further analysis (see Supplementary Fig. S1). The education level of controls was categorized in the same way as for the DACHS participants into ≤ 9 (46.1%), 10 (21.2%), or 12/13 (28.9%) school years. Since controls had completed higher education more often, we adjusted the following analyses for education level. The matching achieved same sex and age-specific distributions in controls as presented in Table 1 for the rehabilitants.

#### HRQOL deficits in rehabilitants and non-rehabilitants

Sex and education-adjusted means of all EORTC-QLQ-C30 scales are presented by age groups for rehabilitants, non-rehabilitants, and controls separately in Supplementary Figs. S2 and S3. Table 5 shows adjusted LSMD between rehabilitants and controls as well as adjusted LSMD between non-rehabilitants and controls by age group. Irrespective of age and past ICR, CRC survivors presented higher scores for both digestive-related scales compared to controls. Deficits were smaller for constipation (up to ten points) than for diarrhea (up to 25 points). Moreover, we observed poorer social functioning (by up to 16 points) and higher financial burden (by up to 12 points) in younger survivors of both treatment groups. However, all deficits were considerably more pronounced in younger rehabilitants. Older rehabilitants and non-rehabilitants of both age groups presented little less pain symptoms and showed comparable HRQOL scores to same-aged controls besides the mentioned deficits. Younger rehabilitants, on the other hand, showed HRQOL deficits in every scale except the dimensions pain and appetite loss.

**Table 5:** Differences in HRQOL (EORTC-QLQ-C30 scores) between CRC survivors and population controls stratified by age

	Age at follow-up/survey < 70 years Comparisons adjusted for sex and education		Age at follow-up/survey ≥ 70 years Comparisons adjusted for sex and education	
	CRC rehabilitants vs population controls	CRC non-rehabilitants vs population controls	CRC rehabilitants vs population controls	CRC non-rehabilitants vs population controls
	Mean Diff. (95% CI)	Mean Diff. (95% CI)	Mean Diff. (95% CI)	Mean Diff. (95% CI)
Function scales^1^				
Physical	− **4.7 (− 7.6, − 1.9)**	− 2.1 (− 5.1, 0.9)	− 1.5 (− 5.1, 2.0)	0.1 (− 3.3, 3.5)
Role	**− 9.7 (− 14.1, − 5.3)**	− 1.7 (− 6.3, 3.0)	− 2.5 (− 7.0, 2.1)	1.8 (− 2.6, 6.2)
Emotional	**− 4.8 (− 8.7, − 0.8)**	0.5 (− 3.7, 4.6)	**− 4.1 (− 7.5, − 0.7)**	− 0.7 (− 4.0, 2.6)
Cognitive	**− 6.4 (− 9.8, − 3.0)**	1.8 (− 1.8, 5.4)	− 2.5 (− 5.9, 1.0)	1.1 (− 2.2, 4.3)
Social	**− 16.5 (− 21.0, − 12.0)**	**− 7.2 (− 11.9, − 2.4)**	− 3.1 (− 7.4, 1.2)	− 1.1 (− 5.2, 3.0)
Global QOL	**− 4.1 (− 7.6, − 0.6)**	2.3 (− 1.4, 6.0)	0.1 (− 3.1, 3.3)	1.8 (− 1.2, 4.8)
Symptom scales^2^				
Insomnia	**6.6 (1.5, 11.7)**	− 0.9 (− 6.3, 4.4)	4.0 (− 0.5, 8.6)	− 1.0 (− 5.3, 3.4)
Fatigue	**5.7 (1.5, 9.8)**	− 0.6 (− 4.9, 3.8)	2.9 (− 0.9, 6.8)	− 2.2 (− 5.9, 1.5)
Pain	− 0.3 (− 4.8, 4.2)	**− 7.2 (− 11.9, − 2.4)**	**− 7.2 (− 11.5, − 2.8)**	**− 10.9 (− 15.1, − 6.7)**
Dyspnea	**4.5 (0.3, 8.7)**	1.9 (− 2.6, 6.3)	3.5 (− 1.0, 8.0)	− 0.5 (− 4.8, 3.9)
Constipation	**10.0 (6.3, 13.8)**	**4.1 (0.2, 8.0)**	**6.4 (2.6, 10.2)**	**5.4 (1.7, 9.0)**
Diarrhea	**25.2 (20.8, 29.5)**	**19.6 (15.0, 24.1)**	**15.2 (11.4, 18.9)**	**11.9 (8.4, 15.5)**
Appetite loss	1.5 (− 1.5, 4.4)	0.1 (− 3.00, 3.2)	2.2 (− 1.0, 5.4)	− 0.7 (− 3.8, 2.4)
Nausea and vomiting	**2.1 (0.1, 4.2)**	1.4 (− 0.7, 3.6)	1.2 (− 0.8, 2.9)	− 0.5 (− 2.2, 1.3)
Financial impact	**12.5 (7.9, 17.1)**	**6.1 (1.3, 10.9)**	1.9 (− 1.5, 5.3)	− 0.7 (− 4.0, 2.6)

Significant mean differences are printed in bold

*CI* confidence interval; *CRC* colorectal cancer

^1^Higher scores indicate better functioning/negative differences indicate poorer functioning compared to population controls. Missing values for every scale < 1% within both studies

^2^Higher scores indicate higher symptom burden/positive differences indicate higher symptom burden compared to population controls. Missing values for every scale < 1% within both studies

## Discussion

Five years after CRC diagnosis, we observed differences in several health-related outcomes between former rehabilitants and non-rehabilitants. Our results indicate higher physical and psychosocial disease burden but also higher posttraumatic growth among rehabilitants. Differences were generally more pronounced among younger than among older CRC survivors. In younger survivors, past ICR was also associated with distress. When compared to population controls, younger survivors of both treatment groups reported deficits with respect to social functioning and financial impact. Irrespective of age and treatment groups, survivors experienced higher levels of bowel dysfunctions. Apart from these deficits, non-rehabilitants and older rehabilitants showed HRQOL scores comparable to population controls, whereas younger rehabilitants experienced deficits in almost every HRQOL dimension.

### Differences in patient-reported outcomes between rehabilitants and non-rehabilitants

At first glance, lower levels of HRQOL and higher levels of distress among rehabilitants in comparison to non-rehabilitants may appear surprising, since a previous analysis from the DACHS study had suggested higher survival rates of patients who had undergone ICR (Scherer-Trame et al. 2021). Furthermore, previous, albeit much smaller studies had reported increases in HRQOL of CRC patients during ICR (Lamprecht et al. 2017; Riedl et al. 2017; Singer and Schulte 2009), even though positive changes in health-related outcomes generally seemed to wane over time (Allgayer et al. 2005, 2012; Biskup et al. 1994; Mehnert et al. 2013). However, respective studies also lacked a control group not undergoing ICR, and study results on treatment benefits on psychological distress are not conclusive (Klocker et al. 2018; Lamprecht et al. 2017; Riedl et al. 2017; Ross et al. 2015). Given the cross-sectional assessment of the patient-reported outcomes in our study, the analysis does not allow to draw any conclusions on potential causal relationships. In particular, despite comprehensive adjustment for a large number of potential confounders, there may still be relevant residual confounding by unmeasured covariates. More specifically, rehabilitants and non-rehabilitants likely differ by factors related to indication of ICR, which are hard if not impossible to completely adjust for. Indication-related health conditions possibly persist or recur after rehabilitation and may influence survivors’ quality of life several years after diagnosis. Finally, selective survival of rehabilitants and non-rehabilitants could also have led to apparent differences in patient-reported outcomes among 5-year survivors. Poorer health outcomes including general health problems, wellbeing, participation, and vitality among CRC survivors who had undergone ICR have been reported previously by Deck et al. (2019). Respective differences between rehabilitants and non-rehabilitants, however, were unadjusted and based on a small sample.

Although the aforementioned factors prohibit drawing firm conclusions, the patterns we observed may give rise to a number of hypotheses that might be worth to be followed up in future research. One such hypothesis would be that rehabilitants might be more health conscious and more alert with respect to detriments in HRQOL or symptoms, and such conscious perception might even be strengthened during ICR. Another hypothesis along similar lines of reasoning might be that ICR may support the development of posttraumatic growth, taking into account that a salutogenetic consciousness, the conveyance of which is a principle of rehabilitation care, and promotes self-care and constructive coping with the disease (Ochoa Arnedo et al. 2019). In addition, higher perceived burden of CRC diagnosis, which was shown to be related to posttraumatic growth (Jansen et al. 2011b), might have led to the decision of undergoing ICR. Nevertheless, for the time being, such hypotheses remain speculative. The final answer on potential effects of ICR on long-term patient-reported outcomes of CRC patients would have to come from randomized controlled trials (RCTs). Although a few mostly small controlled trials have evaluated short-term effects of different treatment modalities among rehabilitants (Allgayer et al. 2004, 2008), to our knowledge, no RCT to date on an intervention comparable to the multidimensional rehabilitation programs offered in Germany has compared long-term outcomes of rehabilitants and non-rehabilitants.

### ***Deficits*** in HRQOL*** in comparison to population controls***

The HRQOL deficits we observed in this cohort of CRC were consistent with previous findings from other studies (Arndt et al. 2017; Caravati-Jouvenceaux et al. 2011; Jansen et al. 2011a; Thong et al. 2019). CRC survivors of both treatment and age groups reported higher levels of bowel dysfunctions and younger survivors generally experienced detriments in social participation and higher financial strain. Contrary, the lower pain levels we observed among this cohort of survivors are in line with findings by Thong et al. (2019) and might be explained by a response shift that reflects a general higher threshold of pain perception and tolerance following cancer treatment (Visser et al. 2013). However, in addition to the confirmation of previous results, we were able to identify a group of CRC survivors—younger rehabilitants—who seem to experience deficits in almost every dimension, whereas non-rehabilitants and older rehabilitants showed HRQOL scores comparable to population controls apart from the aforementioned scales. Since employed patients were more likely to receive ICR than retired patients (Scherer-Trame et al. 2021), poorer cognitive, role functioning, and larger financial strain in younger rehabilitants could derive from an unsuccessful return to work as described in breast cancer patients (Schmidt et al. 2019). Larger deficits in social participation might be linked to higher level of digestive dysfunctions as we have found a significant moderate negative correlation between the scales social functioning and diarrhea for both treatment groups (*r* = − 0.30/− 0.33). Gas and stool leakages and the need for a restroom within reach can affect mobility and social participation severely (Rothbarth et al. 2001). Deficits with respect to emotional and cognitive functioning as well as higher levels of fatigue and insomnia suggest a symptom cluster that indicates higher psychological strain among younger CRC survivors that had undergone ICR.

Younger CRC patients who had undergone ICR may benefit from follow-up concepts after ICR. Follow-up programs aim to maintain treatment success, promote the return to work, and support the rehabilitants applying recommendations into daily life (German Pension Insurance 2019; Koch and Bergelt 2019). However, uptake of these programs is rather low in cancer rehabilitants compared to other indication groups. In recent years, the implementation of telephone- or web-based follow-up concepts has been discussed and tested as pilot projects, since this approach could potentially facilitate nationwide access as well as cost-effective and indication-specific care (Sewöster et al. 2014; Zwerenz et al. 2017).

### Strengths and limitations of the study

We were able to assess differences with respect to past ICR in a large representative cohort of survivors 5 years after CRC diagnosis. Major strengths of the study include comprehensive confounder adjustment, satisfying completeness of data and the high response rate among the DACHS follow-up participants. Non-responders did not differ regarding sex or cancer stage, but were found to be older, lower educated and to have a higher comorbidity score. However, differences seemed to be minor, and ICR utilization was similar among non-responders (44%) and responders (49%). Valid information on possible repeated ICR stays and on ICR that was administered 3 years after diagnosis was not available. Thus, findings refer to any ICR within the postulated period. Standardized cancer-specific instruments were administered to measure HRQOL and distress, but the cross-sectional assessment did not allow us to draw any conclusions on effectiveness of treatment. Longitudinal assessment of the outcomes and relevant information on medical indication for ICR would be prerequisites to evaluate effectiveness.

We identified HRQOL deficits in rehabilitants and non-rehabilitants in comparison to the general population. In addition to the confirmation of previous findings concerning deficits in CRC survivors, we were able to point out the disease-related health burden in younger survivors who had undergone ICR. The response rate within the LinDE study was low which is common for population-based surveys. Therefore, the possibility has to be kept in mind that healthier individuals were more likely to participate. Since this also might be true for the recruitment of the DACHS study or the participation in the follow-up, observed deficits may still not be overestimated. Education levels were higher among the population controls, but this difference could be adjusted for in the analysis. Despite some limitations, the LinDE study served as a very useful population-based reference due to application of the same questionnaire instruments and conduction during the same calendar years.

### Conclusion

The utilization of ICR predicted worse HRQOL scores in several dimensions and distress but also higher posttraumatic growth among CRC survivors. Differences by treatment groups were pronounced in survivors, who were younger than 70 years at follow-up. We found younger rehabilitants to experience strongest deficits of functioning and highest symptom burden when compared to age-matched pairs from the general population. Observed long-term deficits possibly reflect the initial indication for past ICR. Future studies of controlled design and longer observation period are essential to investigate long-term effectiveness of rehabilitation treatment. To optimize treatment allocation and content, further research on determinants of therapy success including potential follow-up concepts after ICR is needed.

## Supplementary Information

Below is the link to the electronic supplementary material.

Supplementary file1 Supplementary Fig. S1 Flow diagram: selection of study participants and population controls (TIF 866 KB)

Supplementary file2 Supplementary Fig. S2 Sex- and education adjusted mean EORTC-C30 functioning scales scores of colorectal cancer survivors by treatment and population controls, stratified by age at follow-up/survey (TIF 1990 KB)

Supplementary file3 Supplementary Fig. S3 Sex- and education adjusted mean EORTC-C30 symptom scales scores of colorectal cancer survivors by treatment and population controls, stratified by age at follow-up/survey (TIF 425 KB)

## Data Availability

Data analyzed for this publication are not publicly available, but will be shared by the corresponding author upon reasonable request from qualified researchers.
