# Supplementary figures and images for: Quality of life, distress, and posttraumatic growth 5 years after colorectal cancer diagnosis according to history of inpatient rehabilitation
Source: J Cancer Res Clin Oncol. 2021 Dec 7;148(11):3015–28. doi: 10.1007/s00432-021-03865-3 (PMC9508041; doi:10.1007/s00432-021-03865-3)

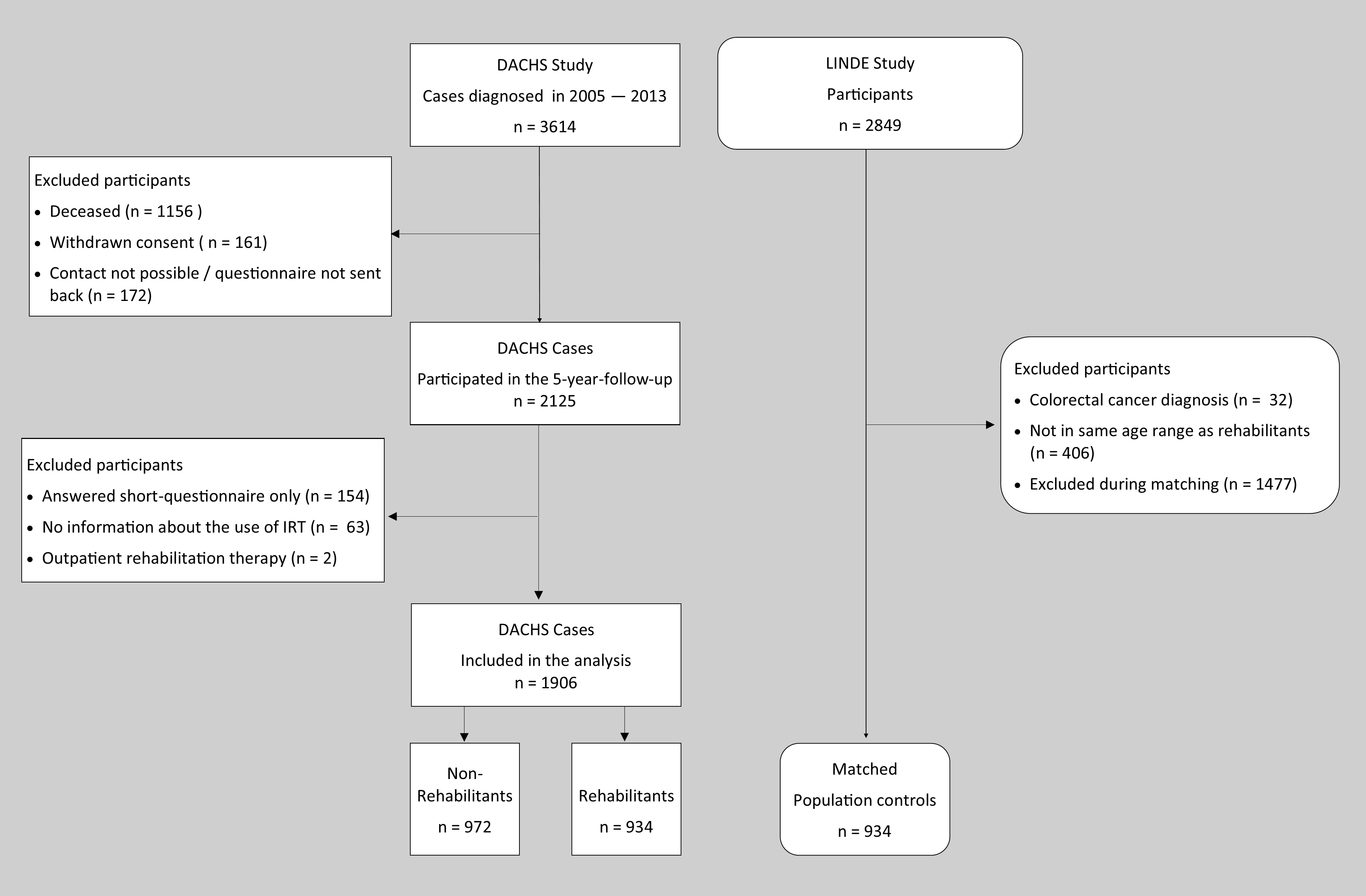

Supplement: Supplementary file 1 — Supplementary file1 Supplementary Fig. S1 Flow diagram: selection of study participants and population controls (TIF 866 KB) [file 432_2021_3865_MOESM1_ESM.tif]

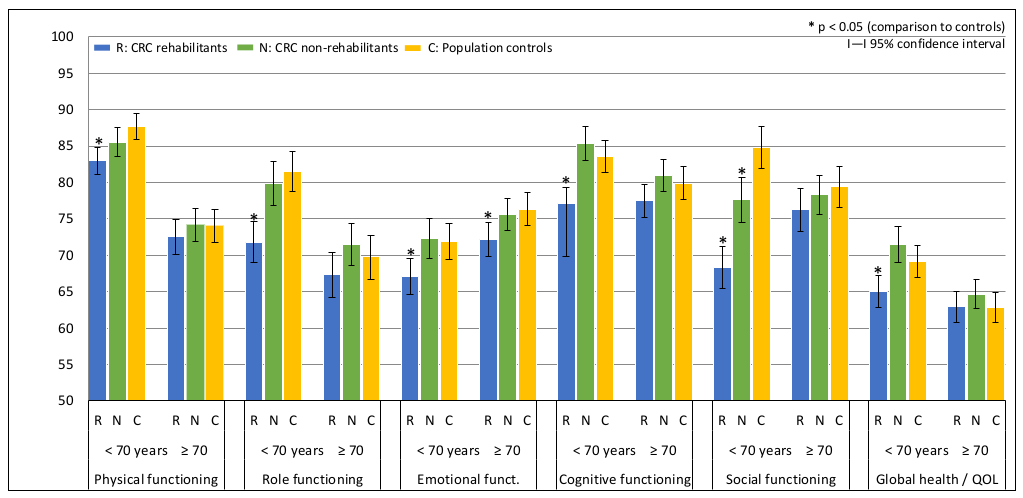

Supplement: Supplementary file 2 — Supplementary file2 Supplementary Fig. S2 Sex- and education adjusted mean EORTC-C30 functioning scales scores of colorectal cancer survivors by treatment and population controls, stratified by age at follow-up/survey (TIF 1990 KB) [file 432_2021_3865_MOESM2_ESM.tif]

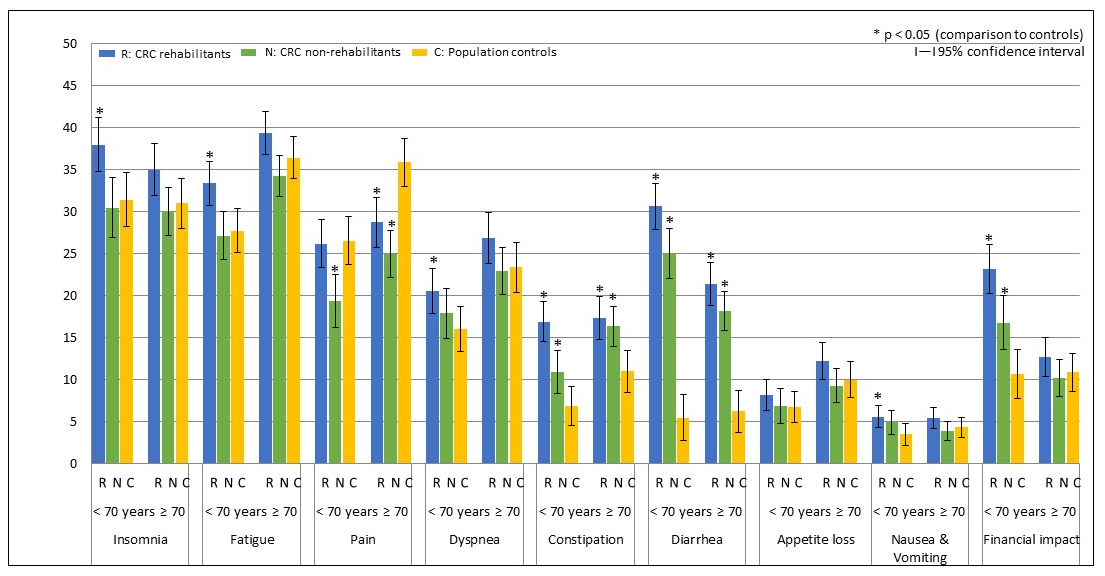

Supplement: Supplementary file 3 — Supplementary file3 Supplementary Fig. S3 Sex- and education adjusted mean EORTC-C30 symptom scales scores of colorectal cancer survivors by treatment and population controls, stratified by age at follow-up/survey (TIF 425 KB) [file 432_2021_3865_MOESM3_ESM.tif]
